# β-Lactamases and β-Lactamase Inhibitors in the 21st Century

**DOI:** 10.1016/j.jmb.2019.04.002

**Published:** 2019-08-23

**Authors:** Catherine L. Tooke, Philip Hinchliffe, Eilis C. Bragginton, Charlotte K. Colenso, Viivi H.A. Hirvonen, Yuiko Takebayashi, James Spencer

**Affiliations:** School of Cellular and Molecular Medicine, University of Bristol Biomedical Sciences Building, University Walk, Bristol BS8 1TD, United Kingdom

**Keywords:** CTX-M, cefotaximase, DBO, diazabicyclooctane, ESBL, extended spectrum β-lactamase, KPC, *Klebsiella pneumoniae* carbapenemase, MBL, metallo-β-lactamase, NDM, New Delhi metallo-β-lactamase, OXA, oxacillinase, PBP, penicillin-binding protein, QM/MM, quantum mechanics/molecular mechanics, SBL, serine β-lactamase, SFX, serial femtosecond crystallography, SHV, sulfydryl variant, TEM, Temoneira (β-lactamase), VIM, Verona imipenemase, β-lactam, antimicrobial resistance, carbapenemase, enzyme mechanism, metallo-β-lactamase

## Abstract

The β-lactams retain a central place in the antibacterial armamentarium. In Gram-negative bacteria, β-lactamase enzymes that hydrolyze the amide bond of the four-membered β-lactam ring are the primary resistance mechanism, with multiple enzymes disseminating on mobile genetic elements across opportunistic pathogens such as Enterobacteriaceae (e.g., *Escherichia coli*) and non-fermenting organisms (e.g., *Pseudomonas aeruginosa*). β-Lactamases divide into four classes; the active-site serine β-lactamases (classes A, C and D) and the zinc-dependent or metallo-β-lactamases (MBLs; class B). Here we review recent advances in mechanistic understanding of each class, focusing upon how growing numbers of crystal structures, in particular for β-lactam complexes, and methods such as neutron diffraction and molecular simulations, have improved understanding of the biochemistry of β-lactam breakdown. A second focus is β-lactamase interactions with carbapenems, as carbapenem-resistant bacteria are of grave clinical concern and carbapenem-hydrolyzing enzymes such as KPC (class A) NDM (class B) and OXA-48 (class D) are proliferating worldwide. An overview is provided of the changing landscape of β-lactamase inhibitors, exemplified by the introduction to the clinic of combinations of β-lactams with diazabicyclooctanone and cyclic boronate serine β-lactamase inhibitors, and of progress and strategies toward clinically useful MBL inhibitors. Despite the long history of β-lactamase research, we contend that issues including continuing unresolved questions around mechanism; opportunities afforded by new technologies such as serial femtosecond crystallography; the need for new inhibitors, particularly for MBLs; the likely impact of new β-lactam:inhibitor combinations and the continuing clinical importance of β-lactams mean that this remains a rewarding research area.

## Graphical Abstract

**Figure:**
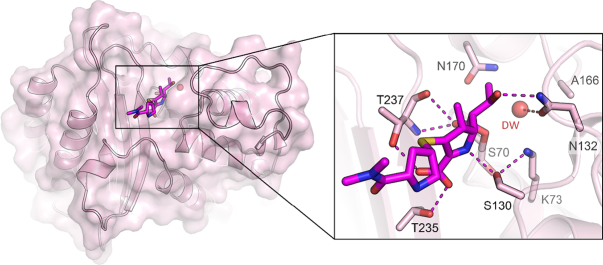

## Introduction

### β-Lactam antibiotics

The discovery of penicillin in 1929 [1] is rightly recognized as a milestone in the history of medicine, and its introduction to the clinic in the 1940s revolutionized our ability to treat bacterial infections. Despite enormous progress in the field of antimicrobial chemotherapy in the more than 70 years after the first use of penicillin, as the centenary of Fleming's work approaches, the β-lactams remain the cornerstones of the antibacterial arsenal. The fact that they remain both the single most prescribed antibiotic class and the most important in terms of sales [2] attests to their continuing central role in the treatment of bacterial infections.

**Fig. 1:**
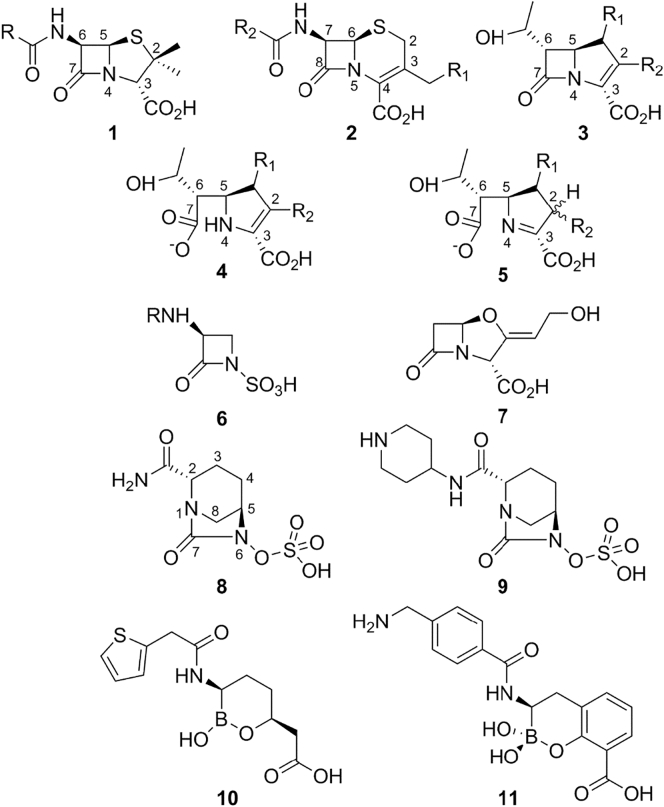
Structures of representative β-lactams and β-lactamase inhibitors. **1**. Penicillin scaffold. **2**. Cephalosporin scaffold. **3**. (1-methyl) Carbapenem scaffold. **4**. Hydrolyzed carbapenem (Δ2-pyrroline form). **5**. Hydrolyzed carbapenem (Δ1-pyrroline form). **6**. Monobactam scaffold. **7**. Clavulanic acid. **8**. Avibactam. **9**. Relebactam. **10**. Vaborbactam. **11**. Bicyclic boronate.

β-Lactams, like other antimicrobial classes, have undergone continuous development since their original introduction in order to improve properties such as potency, spectrum of activity, pharmacokinetic and safety profiles and to counter the emergence of resistance [3]. At present, four main classes of β-lactam antimicrobials are in clinical use (Fig. 1). These comprise three types of bicyclic structure: the penicillins (**1**), in which the four-membered β-lactam ring is fused to a thiazolidine ring; the cephalosporins (**2**), where the fusion partner is a six-membered dihydrothiazine; and the carbapenems (**3**), where the bicyclic system is completed by a five-membered pyrroline. A fourth class, the monobactams (**6**) are monocyclic systems. While each class was originally identified as a natural product (penicillin in 1929, cephalosporins by Newton and Abraham [5] (building on the work of Brotzu [4]) in 1954, olivanic acid (carbapenem) by Brown and co-workers [6] in 1976 and monobactams by Sykes and Imada and their respective co-workers [7], [8] in 1981], each has since undergone extensive programs of modification to create arrays of semi-synthetic derivatives.

The antibacterial activity of β-lactams was identified by Tipper and Strominger [9] as based on their resemblance to the terminal d-Ala–d-Ala moiety of the peptidoglycan stem pentapeptide, with the β-lactam amide and adjoining carboxylate (or, in the case of monobactams, sulfonic acid) groups serving to mimic the peptide bond and terminal carboxylate of d-Ala–d-Ala. Activity then arises from reaction of the β-lactam ring with the nucleophilic serine of target penicillin-binding proteins (PBPs), leading to opening of the ring and irreversible PBP acylation [10] that prevents formation of peptidoglycan transpeptide cross-links. In consequence, modification that allows for retention of antibacterial activity is possible at several positions on the β-lactam scaffold: C6 of penicillins, C7 and C3 of cephalosporins, C2 of carbapenems and C3 of monobactams. Key developments arising from such modifications include introduction of aminopenicillins (e.g., ampicillin) that extended antibacterial activity of penicillins to include Gram-negative bacteria [11]; introduction of methicillin to counter penicillin-resistant strains of *Staphylococcus aureus* [12]; and introduction of oxyiminocephalosporins [13] (e.g., cefotaxime, ceftazidime) to counter emergence of β-lactamase-mediated resistance in Gram-negative bacteria.

### β-Lactamase mediated β-lactam resistance

As with other antimicrobial classes, extensive use of β-lactams has led to the emergence and dissemination of resistance. Resistance can occur by multiple mechanisms, including modification of the target (mutation or expression of alternative PBPs), reduction in cell permeability through downregulation of porins required for β-lactam entry, over-expression of efflux systems and production of modifying or degradative enzymes [14]. In the case of β-lactams, enzyme-mediated resistance arises from the activity of β-lactamases, enzymes produced by both Gram-positive and Gram-negative bacteria that hydrolyze the β-lactam amide [15]. It is not always appreciated that enzymes able to degrade penicillin were identified in *Escherichia coli* even before its first use in man [16], although as initial penicillin use focused largely on infections by Gram-positive pathogens, the significance of this finding was not immediately apparent. β-Lactamases, however, quickly became clinically important as resistance in *S. aureus* arising from production of the PC1 enzyme [17] (encoded by the *blaZ* gene) rapidly compromised penicillin effectiveness. This and the successful countering of PC1-producing organisms through introduction of methicillin [12] heralded the beginning of an “arms race” between medicinal chemists and bacterial evolution that continues to this day and that has seen the introduction of new β-lactams lead to emergence of new β-lactamases both by mutation of previously known families and dissemination of genes encoding new enzymes. Despite the initial importance of PC1-mediated penicillin resistance in *S. aureus*, in Gram-positive bacteria, the emergence of strains carrying PBP alterations has largely superseded β-lactamase production as the primary mechanism of β-lactam resistance [18]. In consequence, this review will largely focus on Gram-negative bacteria, where β-lactamases remain the primary β-lactam resistance mechanism, although it is important to note that in the clinic resistance is very often multifactorial and phenotype is driven by combinations of mechanisms that frequently include permeability modifications and/or efflux pump upregulation in addition to β-lactamase production. A further complication is the increasing prevalence of strains expressing multiple β-lactamases, often encoded upon single multiresistance plasmids (for recent reviews, see Refs. [19], [20]).

### Classification of β-lactamases

Identification of growing numbers of β-lactamases, coupled with availability of protein, and subsequently nucleotide, sequence information, established that these enzymes do not comprise a single homogeneous group but instead can be subdivided into multiple classes. Furthermore, as enzyme activity against different β-lactam substrates began to be reported, it became apparent that β-lactamases encompass a range of biochemical properties. With the explosion of sequence information, the number of identified β-lactamases has undergone a near-exponential increase; at the time of writing, the β-lactamase database (www.bldb.eu [21]) contains over 4300 such enzymes that have undergone varying degrees of characterization.

**Fig. 2:**
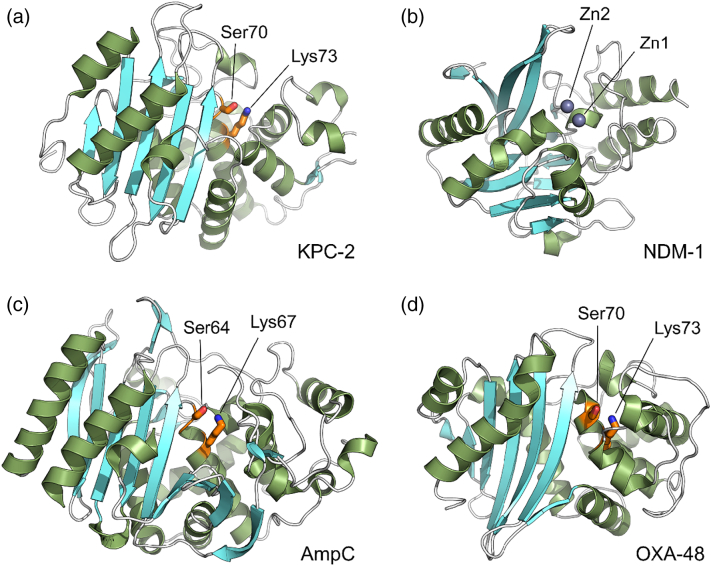
Overall structure of representative β-lactamases from each class. Crystal structures of β-lactamases from classes A, B, C and D. Catalytic important residues of serine-β-lactamases (serine 64/70 and lysine 67/73, labeled) are colored orange, and the metallo-β-lactamase zinc ions are shown as gray spheres. (a) Class A KPC-2 (PDB 5ul8). (b) Class B NDM-1 (PDB 5zgy). (c) Class C AmpC (PDB 1ke4). (d) Class D OXA-48 (PDB 3hbr). Figure (and other structural Figures) generated with Pymol (www.pymol.org).

Two systems of classifying this array of enzymes are in use: the Bush–Jacoby–Medeiros activity-based system [22] and the Ambler system [23] based on sequence information. The latter divides β-lactamases into four distinct classes, termed A, B C and D (Fig. 2), identified on the basis of specific sequence motifs but also distinguished by fundamental differences in hydrolytic mechanism. A further fundamental division is between the three classes (A, C and D) of active-site serine enzymes (seine β-lactamases; SBLs) and class B that comprises a heterogeneous group of zinc metalloenzymes (metallo-β-lactamases, or MBLs). SBLs are distantly related to PBPs [24] (with which they share an invariant Ser–Xaa–Xaa–Lys motif), employ this serine as the reaction nucleophile and hydrolyze β-lactams via a covalent acylenzyme intermediate. MBLs instead utilize a metal-activated water nucleophile to drive the hydrolytic reaction. Although all four classes are widely distributed in multiple species of clinically significant and environmental bacteria, within each class, a few enzyme families have been spectacularly successful and have disseminated widely across the most important bacterial pathogens. These are generally considered to be Gram-negative bacteria responsible for opportunistic healthcare-associated infections of immunocompromised patients, including Enterobacteriaceae such as *E. coli* and *Klebsiella pneumoniae* and non-fermenting species such as *Pseudomonas aeruginosa* and *Acinetobacter baumannii*. Key enzyme families include TEM, SHV, CTX-M and KPC (class A); NDM and VIM (class B); and CMY and ADC (class C). Class D enzymes are all termed oxacillinase (OXA); particular concern surrounds the OXA-23 and 24/40, and the OXA-48 groups, responsible for carbapenem resistance in *A. baumannii* and Enterobacteriaceae, respectively. (It is apparent from the above that the field is considerably complicated by historical but now well-established inconsistencies of nomenclature.)

This review will focus on recent advances in our mechanistic understanding of these and other key enzymes from each of the four Ambler classes, as derived primarily from the growing availability of structural (crystallographic) information describing how β-lactamases interact with their substrates. We will then briefly consider how new developments in the area of β-lactamase inhibitors promise to enable some β-lactamase-mediated resistance to be overcome. A particular interest is the interactions of β-lactamases with carbapenems, as these are increasingly “first-choice” agents for empiric therapy of healthcare-associated Gram-negative infections [25]. In the light of the continuing lack of new antibiotics with anti-Gram-negative activity, carbapenem resistance is a cause of much clinical concern [26], [27].

### Mechanistic overview of serine β-lactamases

**Fig. 3:**
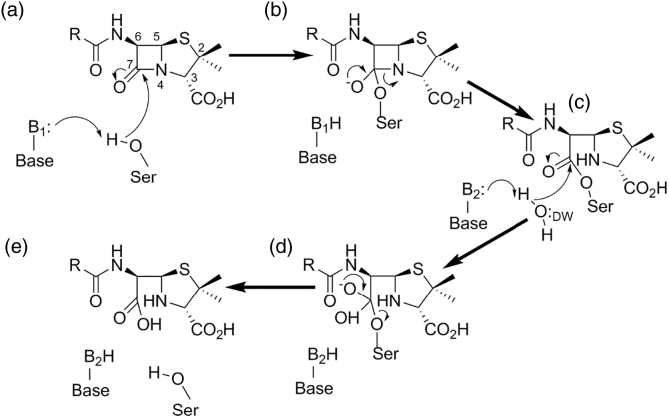
Mechanistic overview of serine β-lactamases. Figure shows hydrolysis of generic penicillin substrate. (a) General base B1 activates Ser for nucleophilic attack on the amide carbonyl carbon (C7) generating covalent acylenzyme (c) *via* tetrahedral oxyanionic acylation transition state (b). General base B2 activates incoming deacylating water molecule DW for nucleophilic attack on the acylenzyme carbonyl liberating penicilloate product (e) via tetrahedral deacylation transition state (d). For clarity, details of proton transfers to N4 and Ser are omitted. Note that the identities of bases B1 and B2 vary between β-lactamase classes.

Although the three classes of SBLs differ substantially in sequence, all employ an acylation–deacylation mechanism, reminiscent of that of serine proteases, in which the nucleophilic serine is activated by a general base, attacks the carbonyl carbon of the scissile β-lactam amide bond and generates the acylenzyme intermediate via a tetrahedral oxyanion transition state (Fig. 3). Resolution of this species occurs when a water molecule, the so-called deacylating water, is activated by a general base for hydrolysis of the acylenzyme and liberation of the degraded product. The three Ambler classes, however, diverge mechanistically, in particular with respect to the identities of, and precise interactions made by, the necessary general base(s). In classes A and C, this represents a long-standing source of debate within the field.

## Class A β-lactamases

The class A enzymes comprise the most widely distributed and intensively studied of all β-lactamases. Notable class A enzymes include PC1, responsible for penicillin failure in *S. aureus*; TEM [28] (named for patient Temoneira), the first plasmid-borne β-lactamase identified in Gram-negative bacteria and active against aminopenicillins and early cephalosporins; SHV (sulfhydryl variant, an enzyme with similar activity to TEM, originally identified on the chromosome of *K. pneumoniae* [29] and subsequently mobilized onto plasmids); CTX-M [30] (cefotaximase, an enzyme inherently active against the oxyiminocephalosporin cefotaxime and that has rapidly disseminated worldwide); and KPC [31] (*K. pneumoniae* carbapenemase). Key to the success of enzymes such as the TEM, SHV and CTX-M families has been their dissemination on plasmids and other mobile genetic elements across a range of Gram-negative pathogens, particularly the Enterobacteriaceae, and their ability to expand their spectrum of activity as new substrates are introduced to the clinic. Thus, acquisition of point mutations that enable TEM and SHV to hydrolyze oxyiminocephalosporins such as cefotaxime and ceftazidime has generated the so-called “extended-spectrum” phenotype (extended-spectrum beta-lactamases, or ESBLs); CTX-M enzymes, which possess some inherent activity against some such substrates, also accumulate mutations to extend their activity and provide resistance to an enlarged range of β-lactams. ESBLs now significantly threaten the continued effectiveness of cephalosporins in a number of clinical contexts [32]. The extensive literature on mutational adaptation in class A enzymes has been recently reviewed by Palzkill [33]; hence, this will not be considered further here. We instead focus on two aspects of class A biochemistry—the continuing quest for understanding of the acylation mechanism and the basis for their wide variation in activity against carbapenem substrates.

### The acylation mechanism of class A β-lactamases

**Fig. 4:**
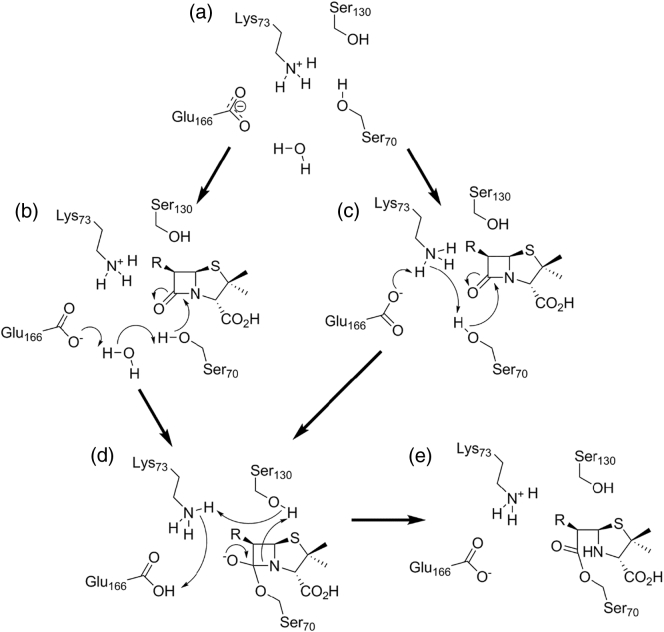
(A) alternative acylation mechanisms for class A β-lactamases. (a) Apoprotein active site shows Lys73 protonated and Glu166 deprotonated. Ser70 may be activated for nucleophilic attack upon the β-lactam carbonyl by Glu166 *via* a water molecule (b). Alternatively, substrate binding reorganizes protonation in the active site such that neutral Lys73 activates Ser70 (c). The transition state (d) resolves to the acylenzyme intermediate (e) by protonation of the amide nitrogen from Ser130.

Despite the wealth of accumulated information, the acylation mechanism of the class A enzymes remains a source of debate and controversy. Two main proposals (Fig. 4) have been put forward, differing in the mechanism by which the serine nucleophile is activated for attack upon the β-lactam carbonyl carbon and, specifically, the identity of the residue acting as the general base in this process. Two conserved residues, Lys73 (part of the SBL SXXK motif) and Glu166, have been proposed to fulfill this function, with both proposals supported by experimental evidence arising from crystallographic studies of enzyme complexes, high-level computational simulations and biochemical investigations of enzyme mutants. Recent contributions in this area stem primarily from a growing availability of high-resolution crystal structures of both free enzymes and covalent complexes, providing direct evidence of protonation states in the β-lactamase active site. These include an increasing number of neutron diffraction studies, made possible by availability of more powerful neutron sources capable of delivering improved diffraction from macromolecular crystals. An attractive prospect is presented by the growing availability of X-ray free electron laser sources that, when coupled with rapid mixing or other reaction initiation technologies, offer the prospect of applying serial femtosecond crystallography (SFX) techniques [34] to capture mechanistically relevant but transiently populated species. The inherently favorable diffraction characteristics of many β-lactamase crystals make these appealing initial targets for application of SFX to studies of enzyme mechanism, the first results of such studies are starting to emerge [35], [36], [37].

Recent evidence supporting involvement of Glu166 as the general base for acylation is provided by both ultrahigh-resolution X‐ray and neutron diffraction studies of the apo-enzymes of CTX-M-9 [38] and Toho-1 [39] (also known as CTX-M-44), which consistently depict active-site hydrogen bonding networks connecting Ser70 and Glu166 via a conserved water molecule and identify Lys73 as present in the protonated form and thus unable to activate Ser70. Moreover, quantum mechanics/molecular mechanics (QM/MM) calculations on TEM-1 [40], [41], [42] support Glu166 as the general base, on the basis that calculated energetic barriers correlate with values derived from experiment and stable species with Lys73 neutral and Glu166 protonated were not observed during the simulations.

The ability of Lys73 to act as general base is supported by the generation of stable acylenzyme complexes, albeit at reduced rates, by mutants at Glu166 [43], [44], consistent with the widely accepted role of this residue as the general base in the deacylation reaction [45], [46], but implying the existence of a viable alternative acylation mechanism. In contrast, alanine mutants of Lys73 have been used to trap non-covalent Michaelis complexes [47], indicative of an essential role for Lys73 in acylation, and high-resolution X‐ray [48] and neutron [49] structures of some acylenzyme complexes of Glu166 mutants show Lys73 to be deprotonated, suggesting that binding of substrate can reduce its p*K*_a_ sufficiently for this to occur. A combined X‐ray/neutron study of a preacylation complex, generated using a CTX-M-44 (Toho-1) Ser70Gly mutant, also reaches this conclusion and is supported by QM/MM calculations of energy barriers for proton transfer from Lys73 to Glu166 *via* Ser70, which reduce in models of the substrate complex compared to the apoenzyme [50]. Similarly, *ab initio* QM/MM calculations [51] have also supported existence of a Lys73-dependent acylation pathway in addition to a Glu166-dependent mechanism. An alternative approach utilizing an analogue of the acylation transition state [52] also indicates proton transfers induced by ligand binding and postulates a low-barrier hydrogen bond to Lys73 as an important contributor to stabilizing the Ser70 nucleophile. Despite these and other advances, however, uncertainties remain: the necessary use of enzyme mutants or substrate analogues in structural characterization of transient species may alter active-site hydrogen bonding networks, while QM/MM simulations remain sensitive to factors including the starting structure/initial model, basis set and QM method used, with consequences for the accuracy of predicted values for energy barriers. Thus, despite substantial recent advances, the acylation mechanism for class A β-lactamases remains to be fully resolved.

### Carbapenem hydrolysis by class A β-lactamases

An area of investigation of class A β-lactamases of much current interest and both mechanistic and clinical relevance concerns the interactions of these enzymes with carbapenems. Carbapenems, introduced to the clinic in 1985 in the form of imipenem [53] (in combination with the renal dipeptidase inhibitor cilastatin required to avoid breakdown *in vivo*), possess both potent antimicrobial activity through inhibition of PBPs, and the ability to effectively acylate, and form long-lived acylenzymes with, the majority of serine β-lactamases. This inhibitory activity encompasses the most widely disseminated class A enzymes of the TEM, SHV and CTX-M families. However, new β-lactamases able to hydrolyze carbapenems have emerged with increasing carbapenem use; in the case of class A, the most important of these are the KPC enzymes [54], which now have worldwide distribution [31]. The mechanistic basis for the activity of these enzymes against carbapenems, which other class A β-lactamases enzymes lack, is attracting continued attention.

**Fig. 5:**
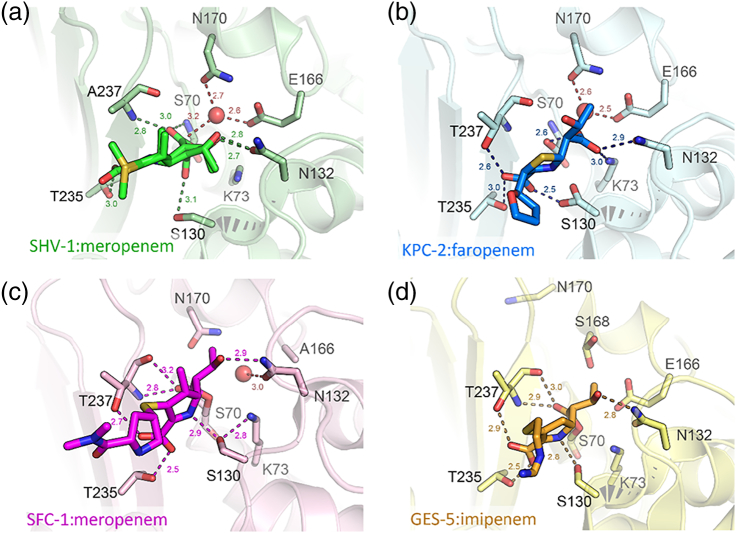
Carbapenem complexes of class A β-lactamases. Close-up views of class A: carbapenem complexes, carbapenem acylenzymes are covalently attached to Ser70. Note hydrogen bonding interactions of the acylenzyme carbonyl with the oxyanion hole formed by the backbone amides of residues 70 (nucleophilic Ser) and 237. (a) SHV-1:meropenem acylenzyme (PDB:2ZD8). Meropenem is modeled in two conformers with the carbonyl oxygen either pointing into the oxyanion hole (interacting with Ala237 and Ser70) or pointing away from the oxyanion hole (interacting with Ser130). In both models, the meropenem R group is not modeled due to disorder. (b) KPC-2:faropenem non-covalent product complex (5UJ4). (c) SFC-1:meropenem acylenzyme (4EV4). (d) GES-5:imipenem acylenzyme (4H8R). Important residues for catalysis and substrate binding (labeled) are represented as sticks, and the deacylating water is displayed as a red sphere. Distances (Å) are displayed as dashed lines.

Carbapenems are distinguished from other β-lactams by the ability of the pyrroline ring to tautomerize between the Δ2 and Δ1 forms (Fig. 1, **4**, **5**), characterized by migration of the double bond from C2=C3 to C3=N. Knowles and co-workers [55], [56] identified that, on reaction with TEM β-lactamase, bound carbapenem, initially in the Δ2 tautomer, could either deacylate or alternatively isomerize to the more inert Δ1 form that accumulated as a stable enzyme-bound species. Subsequent work [57] identified protonation at C3, catalyzed by the Arg244 residue present in TEM-1 and many other class A enzymes, as central to this process. Crystal structures of acylenzyme complexes generated by exposure of class A enzymes to carbapenems (Fig. 5) have in due course described binding modes of both the Δ2 and Δ1 tautomers, but less expectedly also revealed an ability, in the TEM [58] and SHV [59] enzymes, of the acylenzyme carbonyl to migrate between positions within and outside the oxyanion hole formed by the backbone amides of residues 70 and 237. In the case of SHV-1, both of these conformations are observed in a single high-resolution structure (Fig. 5a). Together with Raman crystallographic studies of SHV-1:carbapenem complexes [60], that exploit the distinctive signature of the β-lactam acylenzyme carbonyl to resolve it from bulk protein background, and establish that an initial dual (Δ1/Δ2) population resolves to the Δ1 form, these provide a picture of carbapenem binding in which conformational flexibility of the acylenzyme prevents facile deacylation, enabling isomerization to and accumulation of the stable Δ1 species. An additional key observation is that the 6α-hydroxyethyl substituent of carbapenems adopts positions that enable interaction with elements of the active site important to deacylation, Glu166 in the case of the *Mycobacterium tuberculosis* BlaC enzyme [61] and the deacylating water molecule in SHV-1 complexes [59]. It is then highly likely that such interactions are able to change the pattern of hydrogen bonding in the active site, deactivating the complex for deacylation and instead favoring the competing isomerization reaction.

A central question is then how class A β-lactamases with carbapenemase activity, such as KPC, avoid inhibitory interactions with carbapenems. As structural information on such enzymes became available [62], [63], [64], it was recognized that they are distinguished from other class A β-lactamases by possession of a disulfide bridge between residues 69 and 238, and that their active sites are to some degree expanded compared to those of enzymes inhibited by carbapenems [64], although there are not otherwise gross changes to the active site. Moreover, the importance of the disulfide varies between enzyme families; it appears essential to activity of SME-1 against any β-lactam substrate [65], and its disruption by mutation severely destabilizes the NMC-A and SFC-1 enzymes, but a Cys69Gly mutant of GES-5 is destabilized but remains catalytically competent [66]. These authors conclude that the disulfide is structurally important, but is not an essential and defining requirement for carbapenemase activity.

More recently, crystal structures have become available for carbapenem acylenzymes of the SFC [67] and GES [68] enzymes (Fig. 5, c, d), providing direct observation of carbapenem binding to enzymes with carbapenemase activity. Compared to carbapenem acylenzymes of other class A β-lactamases, these reveal bound carbapenem exclusively in the Δ2, rather than Δ1, form and show that the carbapenem 6α-hydroxyethyl group is oriented to make stable hydrogen-bonding interactions, with Asn132, that preclude deactivating contacts with the deacylating water molecule. The expanded active site of class A carbapenemases may then facilitate this interaction by enabling the necessary rotation about the C6—C7 bond, a conclusion that is supported by molecular dynamics (MD) simulations of SFC-1. In the GES enzymes, where carbapenemase activity is restricted to specific point variants of what are otherwise extended-spectrum enzymes and remains relatively modest, but still clinically relevant, an additional consideration is the longevity of hydrogen-bonding interactions involving Glu166. In GES variants (e.g., GES-5) that hydrolyze carbapenems, persistent H-bonds to Ser170 are proposed, based upon MD simulations, to maintain Glu166 in a position and orientation necessary for deacylation. GES enzymes lacking Ser170, such as GES-1, are unable to hydrolyze carbapenems.

KPC enzymes are by far the most clinically significant of the class A carbapenemases, due to their global distribution in species, principally *K. pneumoniae*, responsible for the majority of opportunistic infections of compromised patients in the healthcare setting [31]. Although structural information on interactions of KPC with β-lactams remains limited, numerous studies have utilized both natural variants [69] and laboratory mutants [70], [71], [72] to investigate the involvement of specific residues in KPC in activity against a range of substrates, including carbapenems. While a carbapenem acylenzyme complex for KPC remains elusive, the structure of a complex with the hydrolysis product of the penem faropenem (Fig. 5b [73]) is consistent with many of these findings, implicating residues such as Pro104, Thr237 and Val240 in substrate binding in the context of an active site that is overall more hydrophobic than that of enzymes such as CTX-M. Nevertheless, high-level computational approaches, typically involving many microseconds of accumulated simulations, have increasingly been applied to study dynamics and conformational transitions in KPC with the aim of establishing a basis for carbapenemase activity. A common finding is that movement of Trp105, a residue that lies on one side of the active site cleft and that adopts multiple conformations in crystal structures of the unliganded enzyme [73], defines transitions between states that differ with respect to their competence for β-lactam turnover. In simulations of carbapenem acylenzyme complexes, committor analysis identifies the conformation of the Trp105 loop as one factor differentiating so-called “permissive” conformations (in which the β-lactam acylenzyme carbonyl occupies the oxyanion hole and the deacylating water is present) from “non-permissive” conformations with the carbonyl outside the oxyanion hole and the deacylating water absent [74]. In multiple-replica parallel tempering metadynamics simulations, that access timescales not available through conventional methodologies, unliganded KPC is shown to undergo motions of active site regions, including Trp105 and the omega-loop containing Glu166, that are related to hydrophobic networks in the individual α and α/β domains and that direct interactions with substrates [75]. Experimental studies of site-directed mutants support these findings by identifying Trp105 as a determinant of KPC activity [71]. QM/MM investigations of carbapenem hydrolysis by class A enzymes, however, remain in their infancy, in part as a result of the relative paucity of crystal structures on which to base starting models. However, a comparison of carbapenem deacylation by a range of class A β-lactamases [76] demonstrated that QM/MM approaches could successfully discriminate between enzymes with carbapenemase activity and acylenzyme systems resistant to deacylation.

When considered together with studies on other class A carbapenemase systems such as those described above, an accumulating body of evidence suggests that activity against carbapenems imposes specific spatial requirements on the class A β-lactamase active site that are necessary to orient bound carbapenems for hydrolysis, but may be more stringent than those associated with other β-lactam substrates. Specifically, a balance may exist between two potentially conflicting requirements: the need to rotate the carbapenem 6α-hydroxyethyl substituent away from the deacylating water and to constrain the acylenzyme carbonyl within the oxyanion hole and prevent access to “non-permissive” conformations. It is likely that multiple structural features, including the conformations of the Trp105 and the omega loops, and the Cys69–Cys238 disulfide bridge, provide the necessary constraints upon the orientation of bound substrates. In this context, it is notable that clinical KPC point variants with increased activity against the (notably bulky) oxyiminocephalosporin ceftazidime exhibit reduced activity against carbapenems [69], indicating a possible association between active-site expansion and reduced carbapenemase activity.

## Class B β-lactamases

**Fig. 6:**
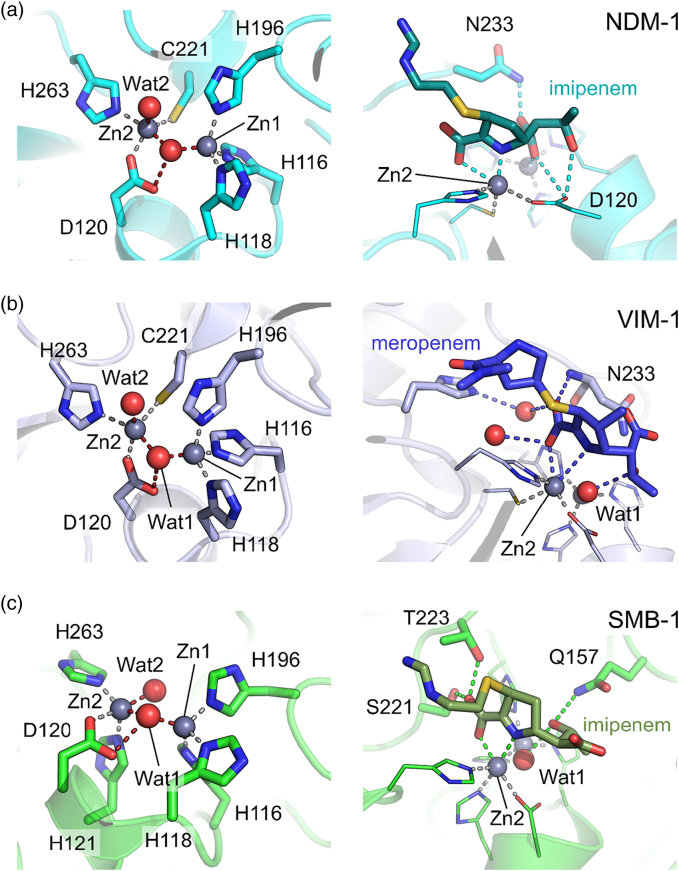
Active-site architecture of class B metallo-β-lactamases and their carbapenem complexes. Close-up views of native metallo-β-lactamase active sites (left) and hydrolyzed carbapenem products (labeled) bound in the active sites (right). (a) B1 NDM-1 (PDBs 5zgx and 5ypk; left and right, respectively). (b) B1 VIM-1 (5n5g and 5n5i). (c) B3 SMB-1 (3vpe and 5b1u).

The class B, zinc-dependent, MBLs are unrelated to known PBPs and instead are members of a large, ancient and widely distributed metallohydrolase superfamily. These enzymes are predominantly found in prokaryotes and act upon a range of substrates extending from various small-molecules (thiolesters, phosphonates) though to nucleic acids [77], [78], and in higher eukaryotes are involved in DNA repair and RNA processing pathways [79]. MBLs are distinguished by a His-Xaa-His-Xaa-Asp motif that forms a metal center located at the interface of the two β-sheets that comprise the core of the protein. It is proposed that the fold originally arose by duplication of an ancestral αβ domain. The identities of the residues that make up this center and its stoichiometry and architecture define three distinct MBL subfamilies (termed B1, B2 and B3). B1 enzymes, the most clinically important, possess a binuclear zinc center comprising tri-His (termed Zn1) and Cys-His-Asp (Zn2) metal sites; in B3 enzymes, the Zn2-coordinating Cys is replaced by an additional His residue; and in B2 enzymes, the first His of the defining motif is replaced by Asn, resulting in a mononuclear enzyme in which only the Zn2 site is occupied [80], [81]. In the binuclear enzymes (Fig. 6), metal coordination is completed by water molecules, one of which (the so-called “bridging” water) connects the two metal ions, and the other (sometimes termed the “apical” water) is bound to Zn2. The geometry of Zn1 is normally considered to be tetrahedral; Zn2 has been described as trigonal bipyramidal or distorted square pyramidal. With the possible exception of the IMP enzymes [82], MBLs are isolated as zinc enzymes, although they are active when reconstituted as other metallated forms (e.g., see Refs. [83], [84], [85]), and other superfamily members utilize a range of other metal ions [77], [79]. As β-lactamases, the MBLs are notable for their exceptionally broad spectrum of activity, which for the binuclear enzymes encompasses penicillins, cephalosporins and carbapenems, although they show little or no activity against monobactams [86], [87].

Compared to other superfamily members and indeed other classes of binuclear metallohydrolases, MBLs exhibit several distinctive features. The presence of Cys, as found in the active site of B1 enzymes, is unusual in catalytic, as opposed to structural, zinc centers [88], as is the absence of any metal-bridging protein group (e.g., a carboxylate side chain), although these are present in other members of the wider MBL superfamily [89]. Understanding of the relationship between the different MBL classes and the wider enzyme superfamily continues to evolve; sequence comparisons indicate that the subclass B3 MBLs are most closely related to other members of the superfamily; and it has been proposed that these are sufficiently distinct from the B1 and B2 MBL subclasses for them to be considered as a separate β-lactamase group [90]. For many years from their identification in 1966 [91], MBLs were considered as primarily of biochemical, rather than clinical, interest, but this situation has changed with the realization that several of the subclass B1 enzymes, notably NDM (New Delhi MBL) in Enterobacteriaceae [92] and VIM (Verona IMipenemase) in non-fermenting organisms, particularly *P. aeruginosa* [93], are disseminating widely on mobile genetic elements. Indeed, the extent of NDM-1 dissemination on a variety of genetic supports has led to its description as an “epidemic gene” and recognition as a major contributor to carbapenem failure in resistant Enterobacteriaceae. In contrast, subclass B2 and B3 enzymes are, with a few exceptions where association of B3 enzymes with mobile genetic elements has been identified [94], [95], restricted to the chromosomes of Gram-negative bacteria. The L1 enzyme of *Stenotrophomonas maltophilia* [96], a highly resistant organism able to infect only severely compromised patients [97], is probably the most clinically relevant of these.

**Fig. 7:**
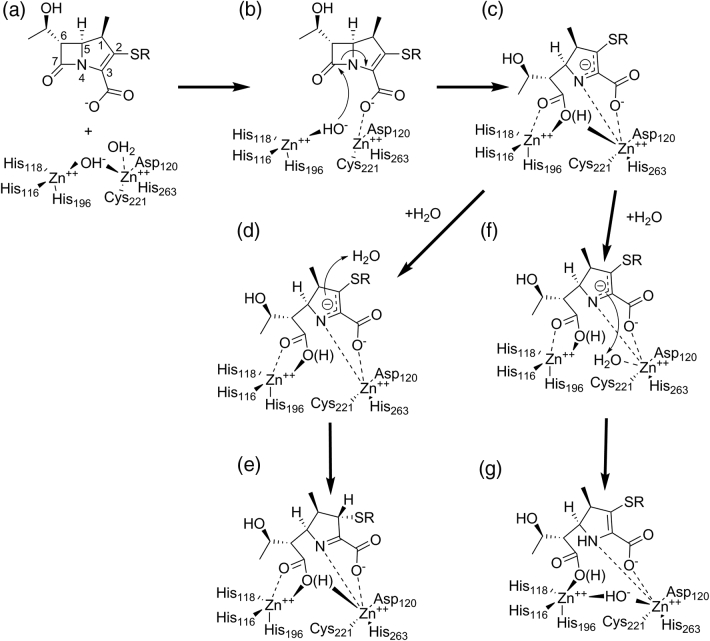
Possible mechanism of carbapenem hydrolysis by binuclear class B metallo-β-lactamases. (a) Substrate binding displaces Zn-bridging hydroxide to a terminal position (b) enabling attack upon the scissile carbonyl. (c) Anionic intermediate with negative charge delocalized around the pyrroline ring resolves either (d) by protonation at C2 by bulk water generating (e) the Δ1 pyrroline or (f) at N4 by incoming water at the bridging position generating (g) the Δ2-pyrroline product. (Note that as shown Zn-bound “apical” water is displaced by substrate; some proposals [115] show this as moving to the bridging position on substrate binding.)

The catalytic mechanism of MBLs (Fig. 7) has now been the subject of extensive investigation, to a great extent facilitated by the ability to substitute zinc for more spectroscopically active metal ions such as cobalt or cadmium, while retaining hydrolytic activity (for review, see Ref. [98]). Against this, the diversity (sequence and structural variation) that exists within even the same MBL subclass complicates the process of identifying the most salient features of β-lactam breakdown that might constitute a common mechanism. A further issue is the long-standing debate over the physiological importance of the binuclear center in B1 MBLs. This arises through the differing affinities of the Zn1 and Zn2 sites (e.g., see Refs. [99], [100]) that permit isolation of forms of the enzymes in which only the Zn1 site is occupied [101], [102], and is complicated by the susceptibility of the Zn2-binding Cys residue to oxidation [103], [104], [105], [106]. The consensus view now holds that the binuclear form of the enzyme is the most catalytically active and predominates under physiological conditions [107]. In consequence, we here focus on mechanistic studies of the binuclear forms of the B1 and B3 enzymes.

### Mechanism of carbapenem hydrolysis by class B β-lactamases

The studies of Waley and Benkovic provide the foundation of much current mechanistic understanding of MBLs. The former proposed, based on low-temperature transient kinetic studies of Zn(II) and Co(II)-substituted enzymes, that reaction involved changes in metal coordination during the catalytic cycle and a branched kinetic mechanism [108], [109]. The latter conclusively supported involvement of a metal-bound water (or hydroxide) nucleophile as opposed to alternatives such as an anhydride mechanism [110]. Benkovic and co-workers also proposed the key intermediate to be a ring-opened (i.e., product-like) anionic species in which a negatively charged nitrogen is stabilized by proximity to Zn2, which is therefore described as a “superacid” [110], [111]. This differentiates the MBL-catalyzed reaction from proposed mechanisms for other metallohydrolases (as reviewed in, e.g., Refs. [112], [113]), which emphasize stabilization by the metal center of tetrahedral oxyanion species formed after attack of a metal-bound water nucleophile on the scissile bond. Although the anionic intermediate was initially identified in hydrolysis of the chromogenic cephalosporin nitrocefin [111], in which formation of a conjugated system within the electron-withdrawing dinitrostyryl C3 substituent is proposed to help stabilize the negative charge, evidence is accumulating for its existence in reactions with other β-lactam classes, in particular carbapenems, where the ability of the pyrroline ring system to tautomerize is considered to support formation of analogous species by enabling delocalization of the negative charge arising from β-lactam amide cleavage [84], [114]. More recently, a combination of UV–vis [utilizing both Zn(II) and Co(II)-substituted enzymes], NMR and X‐ray spectroscopy, together with QM/MM and MD simulations, demonstrated that MBLs of all three classes employ a common, branched, mechanism for carbapenem hydrolysis (Fig. 7), with bifurcation arising from the possibility of protonating the anionic species either at the amide N (with proton donation from metal-bound water at the “bridging” position) or at C2 from bulk solvent [115]. The role of Zn1 is then primarily to deliver the nucleophile, as evidenced by the work of the Page group, who demonstrated that hydrolytic activity of a series of metal-substituted MBL derivatives correlates with the position of the relevant metal ion in the activity series [83].

Accumulation of structural (crystallographic) evidence in support of mechanistic proposals for MBLs has been impeded by the absence of covalent species along the reaction pathway and consequent difficulty in trapping enzyme-bound states. However, several publications [116], [117], [118], [119], [120], [121], [122], [123], [124], [125] now describe structures of enzyme-bound complexes arising from reaction of MBLs with β-lactams *in crystallo*, although (in common with many other crystallographic studies of protein:ligand complexes) the occupancy achieved and the confidence with which the mode of ligand binding is defined vary between them [126]. Complexes of MBLs with hydrolyzed carbapenems have now been described for the B1 enzymes NDM-1 [117], [119], [121] (Fig. 6a) and VIM-1 [125] (Fig. 6b), the B2 enzyme CphA [120] and the B3 enzyme SMB-1 [122] (Fig. 6c). Importantly, the consensus from these structures, as well as those of other complexes of MBLs with hydrolyzed β-lactams [116], [117], [118], [121], [123], supports close approach of the ring-opened nitrogen to Zn2, consistent with the concept of the populated anionic intermediate. However, in other aspects, available structures of carbapenem complexes show considerable variability. A recent study of NDM-1 [119] observed both the Δ2 and Δ1 tautomers of enzyme-bound ring-opened imipenem and meropenem, consistent with the possibility of a branched mechanism, but proposed a linear reaction pathway whereby protonation occurs exclusively from bulk solvent. This was evidenced by NMR experiments which, in contrast to those in the study above, identified only a single diastereomer of the Δ1 hydrolysis product, and by the absence of bound water in the Zn-bridging position, which is instead occupied by the newly formed carboxylate at C7. By way of contrast, carbapenem complexes of VIM-1 [125] (subclass B1) and SMB-1 [122] (B3) exclusively show the Δ1 tautomer, but also contain a water molecule in the bridging position interacting with the 6α-hydroxyethyl group. Notably, these structures were obtained by prolonged exposure to carbapenem rather than the shorter soaks used the NDM-1 study. Taken together, these data suggest that species formed on opening of the β-lactam ring may feature bidentate Zn1 coordination by the product carboxylate, but that an incoming water molecule at the bridging position may cause the complex to reorganize, possibly involving rotation around the carbapenem C5—C6 bond. As in other fields, in the absence of any corroborating information (e.g., spectroscopic information) collected *in crystallo*, assigning crystal structures as specific species in a reaction mechanism defined using solution data remains a challenging undertaking.

Crystallographic studies such as those above have contributed much to our understanding of β-lactam binding and hydrolysis by MBLs, but considerable uncertainties remain. Most notably, structures are lacking for any MBL-bound states in which the β-lactam ring remains intact (i.e., Michaelis complexes) despite efforts to trap these using cadmium-substituted forms of NDM-1 [121]. Information on early stages of the reaction is then derived from spectroscopic studies, in particular the combination of rapid freeze-quenching with electron paramagnetic resonance or X‐ray analysis of fine structure approaches. Such studies, which have provided information at time points down to 10 ms for carbapenem turnover by the BcII (B1) [84], [115] and L1 (B3) [127] MBLs, evidence key aspects of the proposed mechanism for binuclear MBLs, namely, flexible coordination of zinc ions with an increase in coordination number on association with substrate, and flexibility of the metal center as a whole as evidenced by increases in the zinc– zinc separation during the catalytic cycle. The latter point is consistent with crystallographic data [119]. An additional consideration is the existence of specific sequence/structural requirements for turnover of carbapenems that are more stringent than those for other substrates—a very recent deep sequencing study of NDM-1 [128] identified a number of positions at which mutation affected carbapenem hydrolysis much more severely than that of other substrates.

Computational studies of β-lactam binding and hydrolysis by MBLs are similarly constrained by the absence of experimental structures for the Michaelis complex, as well as by the challenges associated with accounting for the flexibility of coordination geometry that is a feature of Zn(II) metalloenzyme systems [129]. In consequence, the findings of QM/MM studies of carbapenem hydrolysis by binuclear MBLs vary in several aspects; notably, interactions involving the substrate carboxylate and Zn2, which in some cases are proposed to occur only on formation of anionic species [130], [131]; the identity of the nucleophile, in one study [121] considered to be bulk water rather than the bridging hydroxide; and the protonation route(s) for N4 [131]. It is nevertheless encouraging that in a number of cases [131], [132] simulations both predicted free energy barriers for carbapenem breakdown and the structures of intermediates that closely approach those derived from experiment.

## Class C β-lactamases

β-Lactamases of class C are widely distributed on the chromosomes of many Gram-negative species. Indeed, as the first β-lactamase to be identified, the *E. coli* enzyme occupies a unique position in the history of β-lactamase research. Many of the most important opportunistic Gram-negative pathogens carry chromosomal genes encoding class C enzymes, typically annotated as *ampC*, that under normal conditions are not expressed. However, derepression of these, either as a result of mutation or through induction by specific β-lactams, can lead to high-level expression with consequent elevation of MICs for susceptible β-lactams [133], [134]. The clinical relevance of class C enzymes is further enhanced by the dissemination of certain family members, such as the CMY, FOX and DHA enzymes, on mobile genetic elements in both Enterobacteriaceae and non-fermenting species such as *P. aeruginosa* [135].

### Mechanism of class C β-lactamases

In many respects, the mechanism of class C enzymes has remained enigmatic, in large part due to the lack of clear candidates for residues able to fulfill the role of general base, with consequent uncertainty surrounding both the acylation and deacylation halves of the reaction. In the absence of the Glu166 found in the class A active site, initial crystallographic work [136] inspired the suggestion, based on structural comparisons with trypsin, that Tyr150 (a residue conserved among class C enzymes) could function as the general base for both acylation and deacylation, but such a mechanism would require this to exist as tyrosinate (i.e., in the deprotonated form) throughout the reaction cycle and is not supported either by NMR titration experiments [137] or by subsequent high-resolution crystal structures [138] that identify this residue as neutral (i.e., remaining protonated) at all stages of reaction. Alternative hypotheses then focus on the role of Lys67 from the SBL SXXK motif and/or require involvement of bound substrate. A further important mechanistic distinction from the class A enzymes, identified from early crystal structures [139], is that the deacylating water molecule approaches from the opposite face of the acylenzyme carbonyl, that is, from the β- rather than the α direction. While understanding of class C β-lactamase mechanism remains incomplete, recent advances in this area have exploited the several high-quality crystal structures now available to apply increasingly sophisticated computational approaches to evaluate possible mechanisms.

Computational approaches have been used to investigate both the acylation and deacylation steps of the class C β-lactamase reaction, although most have focused on the latter. Several investigations have focused on the monobactam aztreonam, for which as a poor substrate, the crystal structure of the wild-type acylenzyme complex is available [136]. MD simulations of AmpC and its Michaelis complex with aztreonam in a range of protonation states [140] supported Lys67 as the general base for acylation. A QM/MM study of AmpC acylation by both aztreonam and the good substrate cephalothin [141] (based on crystal structures of the noncovalent complex determined by the Shoichet group [142]) also supported Lys67 acting as the general base, without necessitating any role for substrate groups. Tyr150 was instead proposed to be involved in protonation of the β-lactam ring N atom, acting via either a bound water molecule or the carboxylate group of bound substrate. This mechanism is in agreement with experimental investigations that show Tyr150 mutants to retain activity [143], as well as mechanistic proposals based on crystal structures [142].

**Fig. 8:**
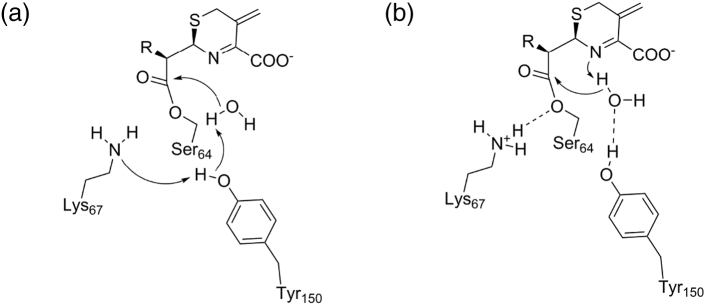
Proposed deacylation mechanisms for class C β-lactamases. Figure shows possible schemes for hydrolysis of generic cephalosporin acylenzyme. (a) Conjugate-base hypothesis where Lys67 deprotonates Tyr150 to activate deacylating water molecule. (b) Substrate-activated hypothesis whereby substrate N deprotonates deacylating water. Dashed lines denote hydrogen bonds.

Proposals for the deacylation mechanism (Fig. 8) now divide largely between those that involve the substrate amide nitrogen (“substrate-assisted”) and those that require coordinated proton transfers between Lys67, Tyr150 and the deacylating water without direct involvement of substrate (“conjugate base”). The latter, first proposed on the basis of QM/MM simulations [144] comparing the *Enterobacter* P99 β-lactamase and the *Streptomyces* R60 DD carboxypeptidase (a model PBP), was apparently disfavored by the relatively small effects upon deacylation of the Lys67Arg mutation [145], but a more recent investigation [146] reported a more substantial loss of activity for this mutant and concluded that the role of Lys67 was more than simply electrostatic. A QM/MM study of deacylation of the good substrate cephalothin [147] identifies Lys67 as responsible for proton transfer to Ser64 in the final stage of the reaction, with Tyr150 undergoing transient deprotonation in the acylenzyme complex that enables its activation for deacylation. These authors emphasize that the dynamic nature of the acylenzyme complex, with frequent proton transfers between these two residues, does not preclude Tyr150 remaining largely protonated in the acylenzyme state. The alternative model for deacylation, in which the catalytic water is deprotonated by the β-lactam ring nitrogen, is supported by crystal structures of acylenzyme complexes [148] and (at higher resolution) of models of the deacylation transition state [138], as well as by the observation that substrate analogues lacking an amine served as irreversible inhibitors [149], but is disfavored by the fact that class C β-lactamases can hydrolyze depsipeptides [150], which also lack an amide nitrogen. It is entirely possible that both mechanisms may be employed, depending on the precise combination of enzyme and substrate [146].

### Carbapenemase activity of class C β-lactamases

**Fig. 9:**
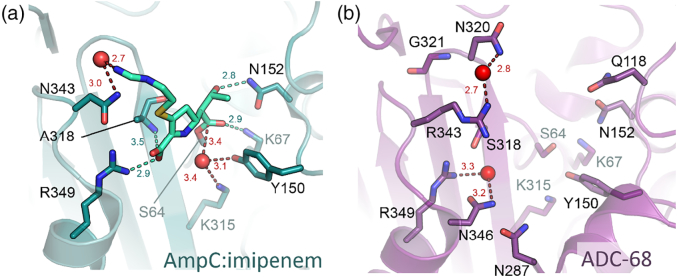
Class C β-lactamase active sites. (a) AmpC:imipenem complex (PDB:1LL5), imipenem acylenzyme covalently attached to Ser64 (note the presence of putative deacylating water adjacent to Tyr150) and (b) ADC-68 active site (note the residues 320 and 321 in the putative C-loop associated with carbapenem turnover). Distances (in Å) displayed as dashed lines. Important residues are represented as sticks (labeled), and waters are shown as spheres.

Class C β-lactamases are not usually regarded as possessing carbapenemase activity, and involvement in carbapenem resistance is generally considered to derive from the ability of strains that combine reduced permeability (i.e., porin loss or mutation) with over-expression of class C enzymes to sequester periplasmic carbapenems before they can reach their PBP targets [151]. Consistent with this, affinities of class C β-lactamases for carbapenems are generally high [152], [153], and the crystal structure of the imipenem acylenzyme formed by *E. coli* AmpC (Fig. 9a [154]) reveals the carbonyl oxygen atom to be rotated approximately 180° away from the oxyanion hole in a binding mode reminiscent of that observed with TEM-1 (see above). Thus, although a water molecule is observed in the putative deacylating position and (due in part to its approach from the β-direction) this is not involved in interactions with the imipenem 6α-hydroxyethyl group, the orientation of the acylenzyme within the active site is considered unfavorable for facile deacylation. Intriguingly, this structure (obtained by relatively short soaking rather than cocrystallization) shows imipenem bound as the Δ2 pyrroline form, with the implication that tautomerization of the carbapenem acylenzyme may occur slowly in class C enzymes compared to some other β-lactamases.

Recently, however, reports have emerged identifying specific class C enzymes as capable of hydrolyzing carbapenems (e.g., imipenem) in addition to extended-spectrum cephalosporin substrates. Crystal structures are now available for two such enzymes, the plasmid-borne CMY-10 originally identified in *Enterobacter aerogenes* [155] and the chromosomal ADC-68 enzyme from *A. baumannii* (Fig. 9b [156]). In both cases, carbapenem turnover is considered to be promoted by a 3-amino-acid deletion in the so-called R2 loop that, by expanding the active site, may relieve potential steric clashes with the extended C2 side chains of many carbapenems. The presence in ADC-68 of glycine, rather than the more bulky arginine found in the parent ADC-1 enzyme (which does not hydrolyze carbapenems), in a second, shorter loop (the C-loop) on the opposite face of the substrate-binding cleft may exert a similar effect [156]. As no acylenzyme structure is yet available for a class C β-lactamase with carbapenem-hydrolyzing activity, it remains unclear whether and how the sequence variations described above also promote deacylation by reorienting the acylenzyme to facilitate attack by the deacylating water molecule.

## Class D β-lactamases

The OXA enzymes of class D are the most diverse and in many respects the least well understood of all the β-lactamases. While the first enzymes identified had activity restricted to penicillins, the OXA class now encompasses enzymes active against cephalosporins and carbapenems and with widely differing sensitivities to inhibitors (for reviews, see Refs. [157], [158]). Although many members are chromosomal, dissemination of plasmid-borne cephalosporinases in *P. aeruginosa* [159], and more recently the spread of carbapenem-hydrolyzing enzymes in *A. baumannii* [160] and in Enterobacteriaceae (particularly *K. pneumoniae* [161]), has increased the clinical significance of this class. The recent identification of OXA enzymes in a variety of Gram-positive species [162] is further indication of the exceptionally wide distribution and diversity of these enzymes.

Although plasmid-borne oxacillin resistance arising from carriage of OXA enzymes was described in the 1960s [28], [163], and OXA enzymes responsible for a range of resistance phenotypes continued to be identified in subsequent years [157], [158], it was not until the turn of the millennium that the first OXA crystal structures became available [164], [165]. The breakthrough in understanding of OXA mechanism arose from the work of Mobashery and co-workers [166], who identified carboxylation of the conserved active-site lysine (the equivalent of Lys73 in class A enzymes) as the key determinant of activity and demonstrated that this arose from reversible reaction with atmospheric carbon dioxide. (It was subsequently shown that acylation of the related BlaR signal sensor responsible for regulating expression of *S. aureus* BlaZ in response to β-lactam challenge is similarly dependent on lysine carboxylation [167], [168].) This unexpected finding (prior to this discovery, carboxylated lysine residues were known only as components of protein metal binding centers) suggested carboxylated lysine as the clear candidate for the general base for both the acylation and deacylation halves of the reaction. This conclusion is supported by subsequent structural work (comparisons with class A enzymes place the OXA lysine carboxylate in a near equivalent position to that of Glu166 [169]), by the inability of lysine mutants to support deacylation [170], and by the results of QM/MM simulations of OXA-23 [171]. The relative hydrophobicity of the OXA active site, which contains markedly more non-polar surface area than the equivalent region of class A β-lactamases [164], is then proposed to contribute to activity by modifying the lysine p*K*_a_ to favor carboxylation. It should, however, be noted that in many [170] but not all [166] cases, lysine mutants retain a residual (though greatly impaired) ability to support acylation (as evidenced by their utility in generating acylenzyme complexes for crystallographic studies). These results indicate that other properties of the OXA active site, besides the presence of the carboxylated lysine, also contribute to activation of the nucleophilic serine.

### Carbapenem hydrolysis by OXA β-lactamases

Much recent effort in the field has concerned the interactions of OXA enzymes with carbapenems, which remains an area of active investigation. Within the greater OXA family, five separate clades of enzymes are generally accepted as being able to hydrolyze carbapenems [158], although it has been proposed, primarily on the basis of their ability to elevate carbapenem MICs in less permeable backgrounds such as *Acinetobacter*, that all OXA enzymes can be considered as carbapenemases [172]. Of the five recognized groups of OXA carbapenemases, four (OXA-23, OXA-24/40, OXA-51 and OXA-58) are primarily associated with resistance in *A. baumannii*, with the OXA-51 group comprising a range of intrinsic, predominantly chromosomal enzymes that can confer resistance when over-expressed as a result of, for example, insertions in promoter sequences [173] and the others more usually associated with plasmids [174], [175]. In contrast, enzymes of the OXA-48 group are found on plasmids in the Enterobacteriaceae [176], where they represent a growing challenge to clinical microbiologists due to their increasingly common involvement in carbapenem failure and the difficulties associated with their detection [161], [177], [178].

**Fig. 10:**
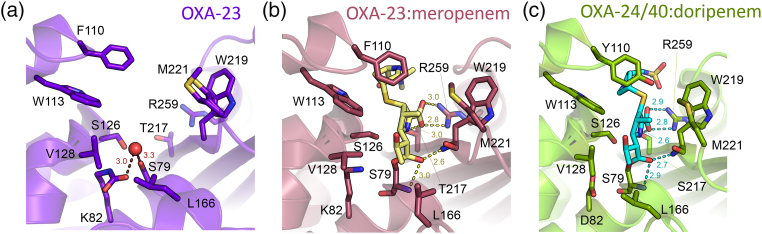
Active sites of class D β-lactamases and carbapenem acylenzymes. (a) Native OXA-23 (PDB 4KOX; note the hydrophobic bridge between Phe110 and Met221 and carboxylated Lys82; deacylating water is shown as a red sphere). (b) OXA-23:meropenem acylenzyme [PDB 4JF4; note the carbapenem acylenzyme (yellow) in Δ1-pyrroline form]. (c) OXA-24/40:doripenem acylenzyme [PDB 3PAE; note the carbapenem acylenzyme (cyan) in Δ2-pyrroline form]. Carbapenem acylenzymes (b and c) shown as sticks covalently attached to Ser79. Distances (in Å) displayed as dashed lines. Note that, for consistency, residue numbering for all panels is as used by Smith *etal*. [185].

Given the diversity within the OXA family, it is perhaps not surprising that variability is evident in the interactions made by carbapenems with OXA carbapenemases. OXA-24 was the first such enzyme for which a crystal structure became available [179]; this revealed a closed tunnel within the active site, created by close contacts between Tyr112 and Met223 on opposite sides of the substrate-binding cleft, that was proposed to orient the carbapenem for hydrolysis *via* interactions with the 6α-hydroxyethyl group. The deleterious effects of mutations at these positions confirmed the importance of these residues to carbapenem turnover. However, a subsequent structure of a doripenem acylenzyme complex of OXA-24 (isolated by mutation of Lys82) suggested instead that Tyr112 and Met223 serve to constrain the orientation of the C3 substituent of bound carbapenem, in so doing favoring retention of the Δ2 configuration for the pyrrolidine ring (Fig. 10c [180]). The Δ2 tautomer is also observed in a doripenem complex of OXA-51 [181], although here the presence of Trp at position 222 (equivalent to Met223) is proposed to impair carbapenem binding [182]. A similar so-called “hydrophobic bridge” between the equivalent residues Phe110 and Met225 is also present in a structure of OXA-58 complexed with the carbapenem analogue 6α-hydroxymethyl penicillin [183], although this is not evident in the uncomplexed enzyme [184] and is thus proposed to form on substrate binding. Uncomplexed OXA-23 (Fig. 10a) also contains an equivalent hydrophobic bridge, between Phe110 and Met 221, but in this case, the structure of a carbapenem complex (here obtained at low pH, rather than by mutation of lysine) showed binding of the Δ1 tautomer (Fig. 10b [185]). Very recently, a more comprehensive mutational study indicated that the effects of substitutions at individual bridge residues on carbapenem turnover are substrate-dependent. Structures of imipenem and meropenem complexes of a double mutant lacking the hydrophobic bridge, however, both revealed the binding of the Δ1 pyrroline, but in the R, rather than S, form. It was concluded that in the wild-type enzyme the bridge residues select against the Δ1-R tautomer via steric hindrance but do not favor the Δ2 over the Δ1-S form [186].

The multiple structural studies described above thus indicate that, while OXA carbapenemases of *Acinetobacter* remain a heterogeneous group, the presence of an active-site hydrophobic bridge represents a common structural feature that is an important contributor to activity toward carbapenems. In contrast, OXA-48 is only distantly related to the *Acinetobacter* enzymes and instead is closer in sequence to enzymes such as the OXA-10 group that are not usually considered as carbapenemases. Consistent with this, the crystal structure [187] shows that OXA-48 lacks an active-site hydrophobic bridge, an observation that may also explain the retention of activity against other substrates (oxacillin) that are poor substrates for other OXA carbapenemase types [188]. However, molecular dynamics simulations of docked carbapenems support a role for some equivalent residues (OXA-48 Thr213 and Arg214) in making interactions with the methyl group of the carbapenem 6α-hydroxyethyl substituent. Furthermore, exchanging the 10-residue loops connecting strands β5 and β6 in OXA carbapenemases (OXA-23, OXA-24 or OXA-48) onto the backbone of the OXA-10 enzyme (not considered a carbapenemase) resulted in acquisition of activity against imipenem [189]. This result suggests that, despite differences in sequence and in the nature of their interactions with bound carbapenem, the β5–β6 loop (that bears, e.g., Met221 of OXA-24) is a determinant of activity against carbapenems across the range of OXA enzymes.

Taken together, these studies identify a number of factors contributing to carbapenem hydrolysis by OXA enzymes. First, structures of carbapenem complexes [180], [181], [185] indicate that the active-site architecture, particularly the hydrophobic bridge, imposes steric constraints upon the orientation and preferred tautomeric form of the carbapenem acylenzyme. Second, evidence both from modeled complexes [187] and experimental structures (in particular the observation of alternative orientations for the 6α-hydroxymethyl group of 6α-hydroxymethyl penicillin in OXA-58 complex structures [183]) also suggests that, as for the class A carbapenemases (see above), specific interactions involving the carbapenem 6α-hydroxyethyl substituent may be important in positioning and orienting the deacylating water molecule. Lastly, it has also been proposed [184], [185] that steric clashes with the carbapenem 6α-hydroxyethyl group may lead to expulsion of water from the active site on acylation by carbapenems, requiring recruitment of a deacylating water molecule from bulk solvent. As a result, carbapenem turnover may also be dependent on access of water to the active site, such that conformational changes in the acylenzyme may be necessary to provide this [185]. Recent observations [190], however, demonstrate that OXA-48-catalyzed carbapenem breakdown can generate lactone species (most likely arising through intramolecular cyclisation of the acylenzyme) alongside the usual hydrolysis products. This indicates that for some (1β-methyl) substrates it is possible to resolve the carbapenem acylenzyme without a requirement for a deacylating water molecule.

## β-Lactamase inhibitors

Alongside improvements to β-lactams themselves, combinations of susceptible β-lactams with mechanism-based β-lactamase inhibitors represent the major strategy for combatting β-lactamase-mediated resistance [191]. Since the discovery and development of clavulanic acid (Fig. 1, **7** [192]) as an irreversible inhibitor of the most widely distributed class A enzymes (e.g., the TEM, SHV and CTX-M classes [193], [194]), penicillin-inhibitor combinations (amoxicillin–clavulanate, ampicillin–sulbactam, piperacillin–tazobactam) have found wide application as treatments for both community- and, particularly, healthcare-associated infections by β-lactamase-producing organisms [195]. However, their limited spectrum of clinically useful activity, which is confined to a subset of class A enzymes that, importantly, does not include KPC [196], coupled with the emergence and dissemination of insusceptible β-lactamase types, necessitates better treatment options for β-lactamase-producing organisms. The search for more widely effective β-lactamase inhibitors is given added impetus by the continued weakness of the antibiotic discovery pipeline for Gram-negative bacteria [197], [198]. Fortunately, in recent years, this process has borne fruit, with representatives of two new inhibitor classes now in the clinic and further compounds and combinations in development.

Space considerations, together with the availability of several excellent recent reviews [199], [200], [201], [202], restrict comments on inhibitors to a relatively brief consideration of some of the most important recent advances. Chief among these is the introduction of the diazabicyclooctanones (DBOs), of which avibactam (formerly NXL-104; Fig. 1, **8**) was the progenitor and first to reach the clinic as a combination with the oxyiminocephalosporin ceftazidime [203]. Avibactam is a mechanism-based non-β-lactam β-lactamase inhibitor, based around a bicyclic core structure, that is able to acylate the active site of serine β-lactamases in a reversible manner [204]. This contrasts with previous (β-lactam-based) inhibitor classes, where acylation is followed by rearrangement and fragmentation events that ultimately lead to irreversible inhibition, as liberation of compound from the active site normally leads to recyclization and regeneration of active inhibitor. Avibactam is a potent inhibitor of β-lactamases of classes A, including KPC enzymes, and C, and the ceftazidime combination is now approved for conditions including complicated intra-abdominal and urinary tract infections, and hospital-acquired and ventilator-associated pneumonias [205]. The success of avibactam has also stimulated development of a number of alternative DBOs, of which the Merck compound relebactam (Fig. 1, **9** [206]) is at the most advanced stage of development as a combination with imipenem. In these compounds, the points of difference occur in the C2, C3 and C4 substituents, which in some cases results in addition of weak antibacterial activity, arising through PBP inhibition, to complement β-lactamase inhibitory action [207], [208], [256].

**Fig. 11:**
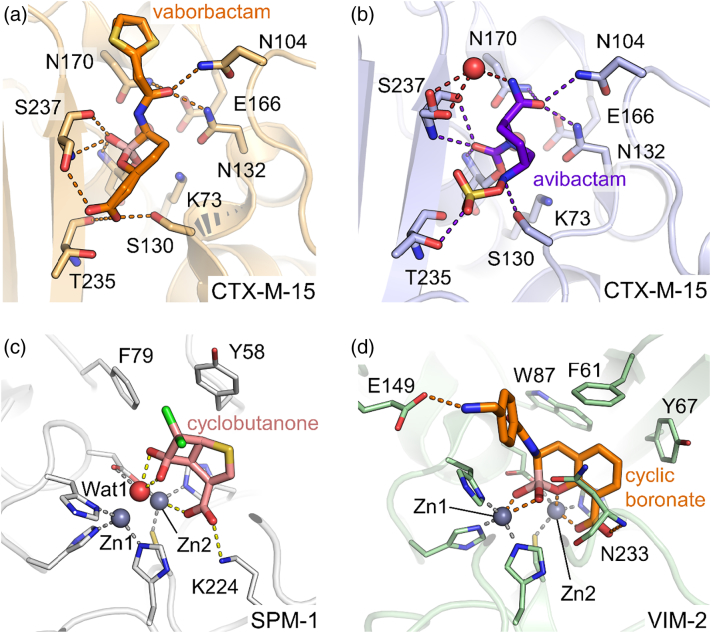
Interactions of β-lactamases with β-lactamase inhibitors. (a) Vaborbactam bound to class A CTX-M-15 (PDB 4xuz). (b) Avibactam bound to CTX-M-15 (PDB 4hbu). (c) A cyclobutanone bound to subclass B2 metallo-β-lactamase SPM-1 (PDB 5ndb). (d) A bicyclic boronate bound to subclass B1 metallo-β-lactamase VIM-2 (PDB 5fqc). Inhibitor molecules and the protein residues they interact, with are shown as sticks. Ligand interactions are shown as colored dashes.

The success of DBOs has driven extensive investigation of their interactions with β-lactamases of multiple structural classes, with the result that inhibition kinetics [207], [209], [210], [211], [212], together with a number of crystal structures [213], [214], [215], [216], [217] (Fig. 11b), are now available for interactions of avibactam in particular with representative enzymes of classes A, C and D. A particular focus of these efforts is the drive to understand determinants of the fate of the acylenzyme complex, which can resolve by recyclization [204] but in some systems, particularly KPC, can also deacylate to generate inactive compound [209]. Evidence from a number of high-resolution structural studies now indicates that the avibactam acylenzyme is able to adopt distinct conformations, differing in the distance between and relative orientation of the N6 and C7 atoms involved in the recyclisation reaction, and whose relative occupancies may provide an indicator of acylenzyme stability [218]. A further complication is the observation of desulfation of the acylenzyme in complexes with KPC, which may be associated with the greater propensity for deacylation in this system [204], [214], [219]. The extent to which modifications of the DBO scaffold and/or mutation of the enzyme target influence these processes is a matter of much current interest. As DBOs have only recently been introduced to the clinic, understanding of the propensity for resistance remains limited, but variants with reduced DBO susceptibility have been generated in the laboratory for both the KPC [219], [220], [221] and AmpC [222], [223], [224] enzymes, and a few reports of KPC mutations in clinical strains unresponsive to DBO therapy have now emerged [225], [226], [227].

Boronate-based compounds represent a second novel scaffold for β-lactamase inhibitor development. The propensity of boron to adopt a tetrahedral geometry enables it to effectively mimic transient tetrahedral species formed during hydrolytic reactions [228], with the result that for some years boronates have found quite extensive application both as inhibitors and as tool compounds in studies of β-lactamase action aimed at mimicking the tetrahedral oxyanion transition states of either the acylation or deacylation reactions (for selected examples, see Refs. [138], [229], [230]). More recently, however, development of cyclic boronates as broad-spectrum inhibitors of serine β-lactamases [231] has resulted in introduction to the clinic of the combination of meropenem with the cyclic boronate vaborbactam (Fig. 1, **10**) as a treatment for complicated urinary tract infections [232]. Vaborbactam potently inhibits β-lactamases of classes A (Fig. 11a and c), but activity does not extend to OXA enzymes or to the MBLs [233].

### Inhibition of MBLs

Introduction to the clinic of DBO and vaborbactam combinations dramatically increases treatment options for serious infections by Gram-negative bacteria. However, neither is effective against the class B (metallo) enzymes, and these now constitute the major challenge for β-lactamase inhibitor development. Indeed, despite extensive academic effort, no MBL inhibitor is yet close to the clinic. The advent of NDM as a highly mobile MBL in Enterobacteriaceae has dramatically increased the clinical importance of MBLs, with the recent observation that MBLs can weakly degrade avibactam (and by implication other DBOs) serving to further emphasize their growing importance. MBL inhibitor development has largely focused on compounds that bind and/or chelate the zinc ions of the active site [200], [201], [202]. The natural compound aspergillomarasmine A inhibits the MBLs NDM-1 and VIM-2 by chelating and removing the active site zinc ions and has been shown to be effective against NDM-1-expressing *K. pneumoniae* in mice [234]. Inhibitors that strongly bind to the active site zinc ions include thiol-based compounds such as the bisthiazolidines, small bicyclic compounds that are effective against B1, B2 and B3 MBLs [235]. Phosphonate-containing compounds (6-phosphonomethylpyridine-2-carboxylates) have also been shown *in vitro* to inhibit B1 and B3 MBLs by interacting with the di-zinc metal center, with inhibition constants in the nanomolar range [236]. More recently, virtual screening combined with NMR resulted in compounds that bound in the active site of VIM-2, but importantly did not ligand the metal ions [237]. To date, the most promising MBL inhibitors are the bicyclic boronates (Fig. 1, **11**), compounds that have been shown to inhibit both SBLs and MBLs (Fig. 11d) by mimicking the tetrahedral oxyanion formed during β-lactam hydrolysis [238]. Their activity extends to NDM-1, and other B1 enzymes, as well as the SBLs CTX-M-15 and OXA-48 [239]. Indeed, a bicyclic boronate (VNRX-5133) in combination with the fourth-generation cephalosporin cefepime is now in phase 1 clinical trials. However, we recently demonstrated that bicyclic boronates were ineffective against the B3 enzyme L1 [240], indicating that their combinations might not be effective against *S. maltophilia*. Identification of compounds such as these with true pan β-lactamase inhibitory activity, that is, able to inhibit both MBLs and SBLs, remains an ultimate goal in the field; the success of cyclic boronates justifies further exploration of β-lactams [241] or analogues such as cyclobutanones [242], [243] (Fig. 11c) where evidence exists that such broad-spectrum activity is also achievable.

Encouraging results have also emerged from recent investigations of alternative approaches to MBL inhibition. The concept of generating covalent inhibitors able to attach to the highly conserved active-site Lys224 of B1 MBLs was first explored by Kurosaki *et* *al*. [244], who demonstrated that Lys224 reacted with activated esters of 3-mercapto propionic acid. More recently, the selenium compound ebselen was shown to attach to Cys221 of NDM-1 [245]; the two approaches have subsequently been combined to create a dual covalent inhibitor, reacting with both Cys221 and Lys224, active against multiple MBLs of subclasses B1 and B2 [246]. A second rewarding approach [247] has been to use colloidal bismuth sulfate (identified from screens of metal compounds) to displace Zn(II) from the NDM-1 active site, generating an inactive Bi(III) complex. Importantly, ebselen and colloidal bismuth sulfate are respectively in trials or clinical use as therapies for other conditions, constituting grounds for optimism that the process of gaining clinical approval may be simpler than for truly novel agents. Despite such progress, inhibition of MBLs remains a challenging undertaking, not least due to the requirement for selectivity over other Zn(II) metalloenzymes within and beyond (e.g., angiotensin-converting enzyme) the MBL superfamily. Achieving potency across the range of MBL enzymes and subclasses is also not trivial; aside from the more obvious divergence of subclass B3 enzymes (as evidenced by the lack of activity of bicyclic boronates against L1) important variation also exists within subclass B1, exemplified by the absence of Lys224 in enzymes such as VIM.

## Concluding Remarks

Several factors have combined to transform the β-lactamase field in recent years. The ubiquity of ESBLs has driven use of carbapenems as empiric therapy for Gram-negative bacteria, leading to dissemination of carbapenemases, particularly in Enterobacteriaceae, that challenge our ability to treat many such infections. In addition to the ongoing proliferation of the KPC enzymes, the association of NDM-1 and OXA-48 with carbapenem resistance in *K. pneumoniae* has provided new impetus for fundamental biochemical studies of β-lactamases of classes B and D, respectively. Furthermore, the explosion in genome sequence information has identified candidate β-lactamases in organisms and environments not previously considered as likely to harbour such enzymes [248], [249], as well as in organisms formerly regarded as niche pathogens that have attracted renewed attention as potential agents of bioterrorism or biological warfare [102], [250], [251], [252].

Fundamental biochemical questions surrounding β-lactamase activity nevertheless remain. For enzymes of classes A and C, debate still surrounds the respective acylation and deacylation mechanisms in particular, while for the metallo-β-lactamases understanding of the early events in the reaction cycle remains far from complete, in no small part due to the absence of any covalent reaction intermediate. The situation is slowly improving as availability of more and better quality crystal structures and increased access to higher-performance computing facilities, respectively, provide more accurate models for mechanistically important species and permit application of high-level computational methodologies to systems of this size and complexity and over biologically more relevant timescales. When coupled with technological advances in fields such as *in crystallo* spectroscopy [253], neutron diffraction and SFX, the prospects for significant advancement in understanding of β-lactamase mechanism are good. Against this, the extensive diversity of both enzyme and substrate in β-lactamase:β-lactam reactions demands caution in extrapolation of results from a single model system to more general conclusions.

The above comments are particularly relevant when considering carbapenem hydrolysis. While the last decade has provided great advances in understanding how enzymes considered as having carbapenemase activity bind and hydrolyze these substrates, questions remain. In particular, the relationship between pyrroline tautomerization and acylenzyme hydrolysis (i.e., the extent and basis of the stability to deacylation of the Δ1 tautomer, particularly in the OXA enzymes) is yet to be established. While the Δ1-pyrroline is observed in many crystal structures of β-lactamase:carbapenem acylenzymes, spectroscopic studies of SHV-1 [60] remain as the strongest direct evidence for its increased stability to hydrolysis compared to the Δ2 acylenzyme. The basis for this slow deacylation remains uncertain: conformational changes associated with formation of the Δ1 species may variously reposition the acylenzyme carbonyl outside the oxyanion hole (SHV-1 [59], [60]), or reorient the 6α-hydroxethyl group to enable interactions that deactivate (e.g., with BlaC Glu166 [61]) or destabilize (e.g., with the carboxylated lysine of OXA enzymes [254]) the deacylating water molecule. A further possibility is that the lack of a proton at N4 of the Δ1-acylenzyme perturbs the active-site hydrogen bonding networks that activate the general base for deacylation [180]. Furthermore, while carbapenemases of classes A and D may both adopt strategies to constrain the orientation of the carbapenem 6α-hydroxyethyl group, the implications of these for binding/activation of the deacylating water molecule may differ in different systems. The interactions of carbapenems with β-lactamases of class C remain much less well understood, and the basis for both carbapenem inhibition of the majority of these enzymes,and carbapenem turnover by exceptions such as CMY-10 and ADC-68 remains unclear.

Aside from continuing investigations of underlying biochemistry, future directions of β-lactamase research will be in large part directed by the emergence and dissemination of resistance phenotypes in the clinic. An open question is then how the overall β-lactamase profile of bacterial pathogens will respond to the clinical application of the new wave of β-lactamase inhibitors. It may be reasonable to predict that MBL-producing strains may become even more prevalent, given that these enzymes continue to avoid inhibitor combinations at or near to clinic, but reports of β-lactamase mutations resulting in insusceptibility to avibactam in clinical isolates carrying KPC [226], [255] or CTX-M [225] enzymes, as well as *in vitro* generation of resistant mutants, demonstrate the existence of multiple potential routes to failure of DBO combinations. Of course, many precedents, including the emergence of KPC and NDM enzymes, also exist to suggest that previously unknown enzymes with distinctive biochemical properties may mobilize in response to usage changes in β-lactams and inhibitor combinations. Identifying the reasons behind the successful large-scale dissemination of enzymes such as those above, when others with similar biochemical properties remain confined to occasional isolates or niche pathogens, may help to estimate the clinical significance of new sequences and enzymes as they continue to be discovered. It is a certainty that the selection pressure imposed by new β-lactams and, particularly, inhibitor combinations will change the landscape of β-lactamases, but much more difficult to predict the ways in which this will occur.

The long history and extensive literature of β-lactamase research might suggest a mature field lacking in scope for discovery science. We would instead argue that the response of the bacterial population to the selection pressure imposed by the continuing clinical importance of β-lactams and of new β-lactam based therapies, the wealth of new enzymes identified through the explosion in microbial sequence information, and the application of new technological advances, together create rich opportunities both for fundamental biochemistry and innovative drug discovery that are underpinned by the pressing need to overcome antimicrobial resistance.
